# Alcohol‐associated liver disease is associated with less adverse outcomes compared to nonalcohol‐associated liver disease in patients with COVID‐19

**DOI:** 10.1111/acer.70124

**Published:** 2025-07-30

**Authors:** Jessica A. Musto, Thomas M. Piasecki, Jeannina Smith, Wendy S. Slutske, Michael C. Fiore, Michael R. Lucey

**Affiliations:** ^1^ Division of Gastroenterology and Hepatology, Department of Medicine University of Wisconsin School of Medicine and Public Health Madison Wisconsin USA; ^2^ Division of General Internal Medicine, Department of Medicine University of Wisconsin School of Medicine and Public Health Madison Wisconsin USA; ^3^ Center for Tobacco Research and Intervention University of Wisconsin School of Medicine and Public Health Madison Wisconsin USA; ^4^ Division of Infections Disease, Department of Medicine University of Wisconsin School of Medicine and Public Health Madison Wisconsin USA; ^5^ Department of Family Medicine and Community Health University of Wisconsin School of Medicine and Public Health Madison Wisconsin USA

**Keywords:** alcohol use disorder, alcohol‐associated liver disease, cirrhosis, mortality

## Abstract

**Background:**

A multicenter cohort of patients hospitalized with COVID‐19 was examined to consider the impact of comorbid liver disease in general, and alcohol‐associated liver disease (ALD) in particular, on short‐term outcomes.

**Methods:**

Data from patients with COVID‐19 hospitalized at 21 participating healthcare systems between February 2020 and January 2022 were examined. The analyses used generalized linear mixed model logistic regression including random intercepts to account for clustering within healthcare systems.

**Results:**

Among 145,944 patients hospitalized with COVID‐19, 7951 (5.4%) had comorbid liver disease; 1153 (14.5%) had ALD, and 6798 (85.5%) had nonalcohol‐associated liver disease (NAALD). The presence of liver disease was associated with increased mortality (adjusted odds ratio [aOR] 3.39, *p* < 0.001), assisted ventilation (aOR 2.95, *p* < 0.001), and ICU admission (aOR 2.27, *p* < 0.001). There was a clear gradient of mortality among the severity of liver disease such that fibrosis < cirrhosis < decompensated cirrhosis. When compared to patients with NAALD, ALD was associated with reduced mortality (aOR 0.36, *p* < 0.001), assisted ventilation (aOR 0.38, *p* < 0.001), and ICU admission (aOR 0.56, *p* < 0.001). On multivariable analysis, liver disease, male gender, increasing age, higher BMI, and former smoking status, but not ALD, were associated with increased mortality with COVID‐19.

**Conclusions:**

In this large cohort of hospitalized COVID‐19 patients, the presence of liver disease increased the odds of all tested adverse outcomes with a mortality gradient that correlated with the severity of liver disease. When compared to liver disease not related to alcohol, ALD was associated with reduced odds of mortality, assisted ventilation, and ICU admission.

## Graphical abstract

This article investigates the connection between liver disease and COVID‐19 outcomes in hospitalized patients. Data were gathered from over 145,000 individuals across 21 healthcare systems over 2 years. Individuals with liver disease generally experienced a higher likelihood of mortality, endotracheal intubation, and ICU admission, with worse outcomes correlating to greater severity of liver disease. However, alcohol‐associated liver disease (ALD) surprisingly showed a reduced risk for these adverse outcomes when compared to other forms of liver disease.

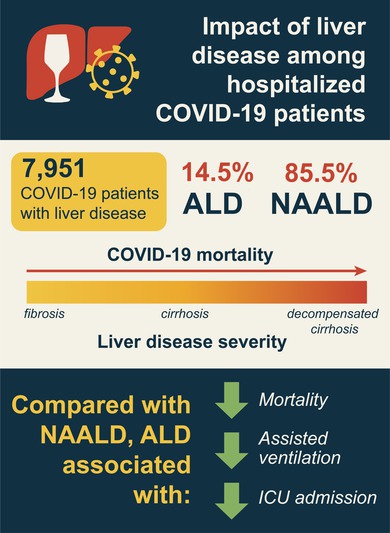

## INTRODUCTION

Prior to the onset of the COVID‐19 pandemic, excessive consumption of alcohol was a serious social problem, with the 12‐month prevalence of alcohol use disorder (AUD) estimated to affect 13.9 million Americans, particularly young adult males, but with rising rates among women (Grant et al., 2015). The COVID‐19 pandemic exacerbated several social stressors that have been implicated as risk factors for increased consumption of alcohol, such as increasing isolation due to mandated “shelter in place” orders, restrictions to communal gathering, financial pressures from loss of employment, and increasing depression (Lindert et al., 2021; Moon et al., 2021). There is evidence that the sale of alcohol in the United States increased during the pandemic, notwithstanding the closure of bars and taverns at various times (Lee et al., 2021). In addition, multiple studies have shown an increase in the prevalence of excessive consumption, including binge drinking during the pandemic (Boschuetz et al., 2020; Weerakoon et al., 2021).

There is a growing body of data to suggest that the burden of care due to alcohol‐associated liver disease (ALD) increased in tandem with the pandemic (Chen et al., 2022; Rutledge et al., 2021). Access to AUD and ALD care were worse than ever; patients with ALD experienced worse outcomes while hospitalized during the pandemic, and mortality rates from ALD increased dramatically (Deutsch‐Link, Curtis, & Singal, 2022; Gao et al., 2023, Sobotka et al., 2023). One study suggested that COVID‐19 infection led to worse outcomes when it occurred in patients with ALD, particularly when accompanied by decompensated cirrhosis or hepatocellular carcinoma (HCC) (Kim et al., 2021). Another found that baseline liver disease and ALD were independent risk factors for death from COVID‐19 (Marjot et al., 2021).

This study examines associations between liver disease and severe COVID‐19 outcomes in a diverse sample 145,944 adults with COVID‐19 infection admitted to 21 hospitals widely distributed across the United States from the beginning of the pandemic (February 2020) to January 2022. It also sought to describe the impact of ALD with COVID‐19. We hypothesized that ALD would correlate independently with adverse consequences of COVID‐19, above and beyond those from liver disease alone.

## MATERIALS AND METHODS

This is a retrospective cohort study of electronic health record (EHR) data collected for the COVID EHR Cohort at the University of Wisconsin (CEC‐UW) https://ctri.wisc.edu/cec‐uw/. Data from all COVID‐19 patients who received care at the 21 participating health systems between February 2020 and January 2022 were examined. A total of 145,944 patients hospitalized with COVID‐19 at 21 participating health systems were included in this study. For patients to be included, the inpatient hospital encounter must have been the first inpatient hospitalization related to COVID‐19, and the duration of the inpatient encounter must have been at least 24 h unless admission to the ICU or death occurred. Patients must have met either of the following: (1) ICD‐10 diagnosis of COVID‐19 assigned during the initial inpatient encounter and/or (2) a positive COVID‐19 PCR test result within a 14‐day period starting 7 days prior to admission through the end of the first week of the initial inpatient encounter. Patients must have had prior contact with the participating health system to permit extraction of pre‐COVID‐19 ICD‐10 diagnoses to calculate the Elixhauser Comorbidity Score (van Walraven et al., 2009). EHRs were queried for ICD‐10 diagnostic codes for alcohol‐associated hepatitis (AH), cirrhosis, and cirrhosis with features of decompensation (ascites, hepatic encephalopathy, variceal hemorrhage, and jaundice). Table S1 in the Supplement shows the breakdown of ICD‐10 codes for each of the patient groups. Unadjusted models contained data for all 145,944 patients. Four patients were excluded from adjusted models due to missing data.

**FIGURE 1:**
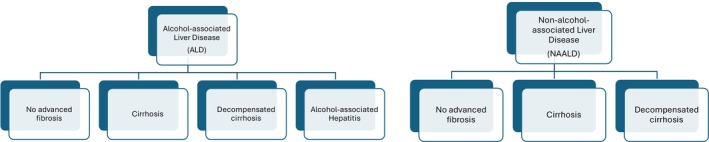
Cohort grouping.

Analysis was performed using four separate approaches: (1) outcomes related to COVID‐19 were compared in those with a history of liver disease of any severity versus those with no history of liver disease; (2) those with liver disease were further subcategorized into 3 severity levels (liver disease without advanced fibrosis, cirrhosis, and decompensated cirrhosis) and outcomes were compared to those with no liver disease; (3) a binary indicator of alcohol‐associated liver disease was added as an independent variable to the analysis with three severity levels of liver disease; and (4) the three severity levels of liver disease were further separated into those with ALD and those with NAALD, and outcomes were compared to those with no history of liver disease (Figure 1). When multiple diagnoses were present, patients were assigned to the highest severity category for which there was a diagnosis present. If a patient had codes that included both ALD and NAALD, they were assigned to the ALD group. I if they had codes that included both AH and ALD of some other category, they were assigned to the AH group.

The primary outcome of interest was overall in‐hospital morality in patients who tested positive for COVID‐19. Secondary outcomes included intubation for mechanically assisted ventilation and ICU admission. Relations between liver disease and hospital outcomes were analyzed using generalized linear mixed model logistic regression analyses incorporating random intercepts to account for the clustering of patients within health systems (Hedeker, 2005). Covariates included sex, age, race, ethnicity, insurance status, smoking status, body mass index, the use of remdesevir and corticosteroids, hospitalization before or after COVID‐19 vaccine availlability, number of COVID‐19 vaccine doses, and a van Walraven‐weighted Elixhauser comorbiditiy index based on a 5‐year look‐back (van Walraven et al., 2009).

There is robust evidence that certain populations of patients are at greater risk for adverse outcomes from COVID‐19 (Bennett et al., 2021; Booth et al., 2021; Garg et al., 2020; Vardavas & Nikitara, 2020). To compare our study's findings to existing knowledge, we also examined COVID‐19 outcomes in known at‐risk populations: those of increasing age, those with a higher BMI, and former smokers.

## RESULTS

### Patient characteristics

A total of 145,944 patients were included in this study. Patients ranged in age from 19 to 90+ years with a median age of 63 years (mean 61.1, SD 18.4 years). Male patients made up 48.9% of the population. A total of 85,851 patients (58.8%) were White, 34,663 patients (23.8%) were Black, and the majority (82.7%) were not Hispanic or Latino. BMIs ranged from underweight (<18.5 kg/m^2^) to severely obese (>40 kg/m^2^) with a median BMI of 29.1 (mean 30.4, SD 8.39 kg/m^2^) and the majority of patients (85.3%) were in the healthy weight (18.5–24.9 kg/m^2^) to obese (30–39.9 kg/m^2^) categories (Table 1, Supplement 2).

**TABLE 1:** Patient characteristics.

	Total (*N* = 145,944)	No of liver disease (*N* = 137,993)	Liver disease (*N* = 7951)	Test
Sex				
Female	74,538 (51.1%)	71,064 (51.5%)	3474 (43.7%)	*χ* ^2^ (2) = 183.61, *p* < 0.001
Male	71,402 (48.9%)	66,925 (48.5%)	4477 (56.3%))	
Other	4 (0.0%)	4 (0.0%)	0 (0.0%)	
Age group				
18–29	9871 (6.8%)	9554 (6.9%)	317 (4.0%)	*χ* ^2^ (6) = 1037.99, *p* < 0.001
30–39	13,051 (8.9%)	12,389 (9.0%)	662 (8.3%)	
40–49	14,666 (10.0%)	13,616 (9.9%)	1050 (13.2%)	
50–64	39,610 (27.1%)	36,632 (26.5%)	2978 (37.5%)	
65–74	29,868 (20.5%)	28,089 (20.4%)	1779 (22.4%)	
75–84	23,984 (16.4%)	23,101 (16.7%)	883 (11.1%)	
85+	14,894 (10.2%)	14,612 (10.6%)	282 (3.5%)	
Race				
American Indian or Alaska Native	546 (0.4%)	485 (0.4%)	61 (0.8%)	*χ* ^2^ (7) = 149.55, *p* < 0.001
Asian	3882 (2.7%)	3674 (2.7%)	208 (2.6%)	
Black or African American	34,663 (23.8%)	33,161 (24.0%)	1502 (18.9%)	
Native Hawaiian or Other Pacific Islander	588 (0.4%)	549 (0.4%)	39 (0.5%)	
White	85,851 (58.8%)	80,953 (58.7%)	4898 (61.6%)	
Other race not specified	17,384 (11.9%)	16,314 (11.8%)	1070 (13.5%)	
More than one race	571 (0.4%)	533 (0.4%)	38 (0.5%)	
Unknown, not reported, or missing	2459 (4.7%)	2324 (1.7%)	135 (1.7%)	
Ethnicity				
Not Hispanic or Latino	120,761 (82.7%)	114,435 (82.9%)	6326 (79.6%)	*χ* ^2^ (2) = 61.48, *p* < 0.001
Hispanic or Latino	22,373 (15.3%)	20,914 (15.2%)	1459 (18.3%)	
Unknown, not reported, or missing	2810 (1.9%)	2644 (1.9%)	166 (2.1%)	
BMI^a^				
Underweight	4504 (3.1%)	4300 (3.2%)	204 (2.6%)	*χ* ^2^ (4) = 129.17, *p* < 0.001
Healthy weight	33,608 (23.3%)	31,978 (23.4%)	1630 (20.6%)	
Overweight	41,473 (28.7%)	39,390 (28.9%)	2083 (26.4%)	
Obese	48,138 (33.3%)	45,306 (33.2%)	2832 (35.9%)	
Severely obese	16,627 (11.5%)	15,482 (11.3%)	1145 (14.5%)	

^a^ BMI data were unavailable for 1594 patients (No liver disease: *n* = 1537; liver disease: *n* = 57).

Among the overall cohort, 7951 patients (5.4%) were recorded to have some form of liver disease; at the time of their hospital admission, 2657 (33.4%) with decompensated cirrhosis, 1666 (21.0%) with compensated cirrhosis, and 3628 (45.6%) with no advanced fibrosis. Those with liver disease were then further categorized into liver disease from an ALD etiology (1153 patients, 14.5%) vs. liver disease from a NAALD etiology (6798 patients, 85.5%). Of those with ALD, 473 (41.0%) had decompensated cirrhosis, 294 (25.5%) had compensated cirrhosis, 271 (23.5%) had liver disease without advanced fibrosis, and 115 (10.0%) had AH.

### Approach #1: Outcomes related to COVID‐19 in those with liver disease vs. no liver disease

The presence of liver disease was associated with increased odds of all tested adverse outcomes in patients hospitalized with COVID‐19 (Table 2). Of those patients with liver disease, 1612 (20.3%) of patients died while hospitalized with COVID‐19 compared to 11,424 (8.3%) without liver disease (aOR 3.39, 95% CI 3.18, 3.62, *p* < 0.001). The presence of liver disease was also associated with increased odds of intubation (aOR 2.95, 95% CI 2.79, 3.12, *p* < 0.001) and ICU admission (aOR 2.27, 95% CI 2.15, 2.38, *p* < 0.001) in both univariate and covariate‐adjusted models.

**TABLE 2:** Observed outcomes by presence or absence of liver disease at index COVID‐19 hospitalization.

Predictor	Unadjusted			Adjusted		
	OR	95% CI	*p*	aOR	95% CI	*p*
Outcome: Mortality						
No liver disease (REF)	1.00	—	—	1.00	—	—
Any liver disease	3.10	2.92, 3.29	<0.001	3.39	3.18, 3.62	<0.001
Outcome: Intubation						
No liver disease (REF)	1.00	—	—	1.00	—	—
Any liver disease	3.12	2.97, 3.29	<0.001	2.95	2.79, 3.12	<0.001
Outcome: ICU admission						
No liver disease (REF)	1.00	—	—	1.00	—	—
Any liver disease	2.44	2.32, 2.56	<0.001	2.27	2.15, 2.38	<0.001

### Approach #2: Outcomes related to COVID‐19 based on liver disease severity

Not only did patients with liver disease have worse outcomes compared to those with no liver disease, but there was also a clear gradient of mortality among the severity of liver disease (Table 3). Those with COVID‐19 and liver disease without advanced fibrosis had increased odds of in‐hospital mortality (aOR 1.36, 95% CI = 1.20, 1.55, *p* < 0.001) compared to those with no liver disease, and mortality only increased with cirrhosis (aOR 2.09, 95% CI 1.81, 2.40, *p* < 0.001) and decompensated cirrhosis (aOR 8.41, 95% CI 7.67, 9.21, *p* < 0.001) on covariate‐adjusted analysis. Liver disease without advanced fibrosis was not significantly different from the no‐liver disease reference group before covariate adjustment.

**TABLE 3:** Observed outcomes by severity of liver disease at index COVID‐19 hospitalization.

Predictor	Unadjusted			Adjusted		
	OR	95% CI	*p*	aOR	95% CI	*p*
Outcome: Mortality						
No liver disease (REF)	1.00	—	—	1.00	—	—
Liver disease no advanced fibrosis	1.09	0.97, 1.23	0.15	1.36	1.20, 1.55	<0.001
Cirrhosis	2.25	1.97, 2.58	<0.001	2.09	1.81, 2.40	<0.001
Decompensated cirrhosis	8.00	7.37, 8.68	<0.001	8.41	7.67, 9.21	<0.001
Outcome: Intubation						
No liver disease (REF)	1.00	—	—	1.00	—	—
Liver disease no advanced fibrosis	1.57	1.44, 1.72	<0.001	1.50	1.37, 1.65	<0.001
Cirrhosis	1.96	1.73, 2.21	<0.001	1.89	1.66, 2.15	<0.001
Decompensated cirrhosis	7.90	7.3, 8.55	<0.001	7.50	6.88, 8.19	<0.001
Outcome: ICU admission						
No liver disease (REF)	1.00	—	—	1.00	—	—
Liver disease no advanced fibrosis	1.44	1.33, 1.56	<0.001	1.39	1.28, 1.50	<0.001
Cirrhosis	1.66	1.49, 1.85	<0.001	1.55	1.38, 1.73	<0.001
Decompensated cirrhosis	5.65	5.22, 6.12	<0.001	5.07	4.66, 5.51	<0.001

Those with COVID‐19 and liver disease without advanced fibrosis also had increased odds of intubation (aOR 1.50, 95% CI 1.37, 1.65, *p* < 0.001) and again these odds increased in cirrhosis (aOR 1.89 95% CI 1.66, 2.15, *p* < 0.001) and decompensated cirrhosis (aOR 7.50, 95% CI 6.88, 8.19, *p* < 0.001). Finally, liver disease was associated with increased odds of admission to the ICU compared to no liver disease (aOR 1.39, 95% CI 1.28, 1.50, *p* < 0.001) and odds increased in cirrhosis (aOR 1.55, 95% CI 1.38, 1.73, *p* < 0.001) and decompensated cirrhosis (aOR 5.07, 95% CI 4.66, 5.51, *p* < 0.001).

### Approach #3: Outcomes related to COVID‐19 based on liver disease severity and binary indicator of alcohol‐associated liver disease

To test further the impact of ALD on COVID‐19 outcomes, we looked at whether liver disease severity and alcohol‐associated etiology were independent predictors of all tested clinical outcomes in patients hospitalized with COVID‐19 (Table 4). Again, each category of liver disease was associated with worse outcomes compared with no liver disease, with the expected severity gradient. In addition, having an ALD diagnosis was associated with reduced odds of adverse outcomes compared to NAALD. Having ALD was associated with reduced odds of mortality (aOR 0.36, 95% CI 0.29, 0.45, *p* < 0.001), intubation (aOR 0.39, 95% CI 0.32, 0.46, *p* < 0.001), and ICU admission (aOR 0.57, 95% CI 0.49, 0.66, *p* < 0.001) compared to NAALD.

**TABLE 4:** Observed outcomes in alcohol‐associated liver disease (ALD) vs. nonalcohol‐associated liver disease (NAALD) at index COVID‐19 hospitalization.

Predictor	Unadjusted			Adjusted		
	OR	95% CI	*p*	aOR	95% CI	*p*
Outcome: Mortality						
NAALD (REF)	1.00	—	—	1.00	—	—
ALD	0.24	0.2, 0.3	<0.001	0.36	0.29, 0.45	<0.001
Outcome: Intubation						
NAALD (REF)	1.00	—	—	1.00	—	—
ALD	0.29	0.24, 0.34	<0.001	0.39	0.32, 0.46	<0.001
Outcome: ICU admission						
NAALD (REF)	1.00	—	—	1.00	—	—
ALD	0.29	0.24, 0.34	<0.001	0.57	0.49, 0.66	<0.001

### Approach #4: Outcomes related to COVID‐19 based on liver disease severity in NAALD versus ALD

**FIGURE 2:**
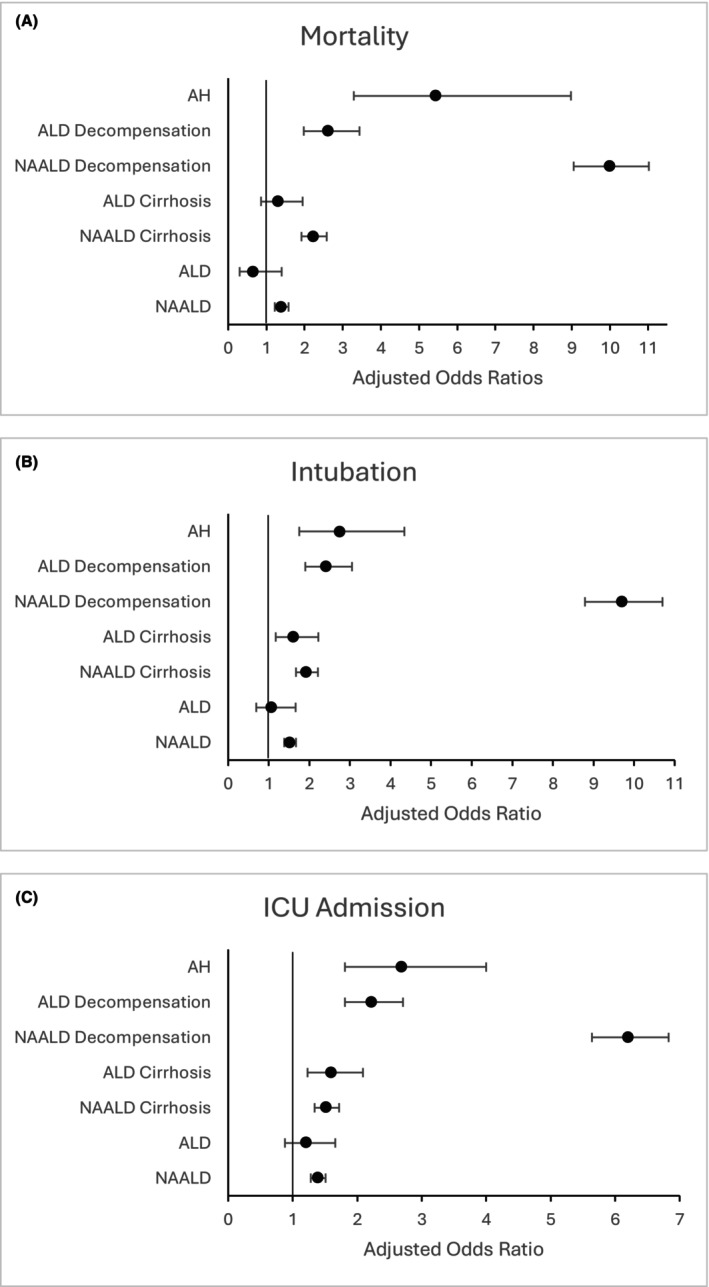
Forest plots of adjusted odds ratios by diagnosis and outcome. Horizontal bars indicated 95% confidence intervals. (A) Mortality. (B) Intubation. (C) ICU admission. ALD, alcohol‐associated liver disease; AH, alcohol‐associated hepatitis; NAALD, nonalcohol‐associated liver disease.

Those patients with liver disease were then further separated into those with NAALD versus ALD versus AH (Figure 1). Most ALD categories were again associated with a higher rate of all adverse outcomes compared to those with no liver disease. However, odds ratios and covariate‐adjusted odds ratios for NAALD were higher than those for comparable ALD. AH was associated with a higher rate of all adverse outcomes compared to ALD of all other categories (Table 5). A severity gradient was observed such that AH > decompensated ALD cirrhosis > ALD cirrhosis > ALD in terms of risk for all tested adverse outcomes. In the unadjusted model, ALD without advanced fibrosis had a significantly decreased odds of mortality relative to no liver disease (OR 0.32, 95% CI 0.15, 0.67, *p* = 0.003), but this effect was no longer significant after covariate adjustment (aOR 0.66, 95% CI 0.31, 1.41, *p* = 0.279). Mortality in the ALD cirrhosis group did not differ significantly from that of patients with no liver disease in univariate and covariate‐adjusted models (aOR 1.31, 95% CI 0.88, 1.97, *p* = 0.188). In comparison, mortality in the NAALD cirrhosis group was statistically significantly higher than that of those with no liver disease on both univariate‐ and covariate‐adjusted models (aOR 2.22, 95% CI 1.91, 2.58, *p* < 0.001). Decompensated ALD cirrhosis was associated with increased odds of mortality (aOR 2.66, 95% CI 2.02, 3.51, p < 0.001) but not to the same degree as AH (aOR 5.83, 95% CI 3.53, 9.63, *p* < 0.001) nor decompensated NAALD cirrhosis (aOR 10.21, 95% CI 9.24, 11.28, *p* < 0.001) (Figure 2A).

**TABLE 5:** Observed outcomes by severity of liver disease in alcohol‐associated liver disease (ALD) versus nonalcohol‐associated liver disease (NAALD) versus alcohol‐associated hepatitis (AH) at index COVID‐19 hospitalization.

Predictor	Unadjusted			Adjusted		
	OR	95% CI	*p*	aOR	95% CI	*p*
Outcome: Mortality						
No liver disease (REF)	1.00	—	—	1.00	—	—
NAALD no advanced fibrosis	1.16	1.03, 1.32	0.016	1.40	1.23, 1.59	<0.001
NAALD cirrhosis	2.48	2.15, 2.85	<0.001	2.22	1.91, 2.58	<0.001
NAALD decompensated cirrhosis	10.70	9.78, 11.71	<0.001	10.21	9.24, 11.28	<0.001
ALD no advanced fibrosis	0.32	0.15, 0.67	0.003	0.66	0.31, 1.41	0.279
ALD cirrhosis	1.28	0.87, 1.89	0.218	1.31	0.88, 1.97	0.188
ALD decompensated cirrhosis	1.96	1.51, 2.54	<0.001	2.66	2.02, 3.51	<0.001
Alcohol‐associated hepatitis	2.89	1.81, 4.62	<0.001	5.83	3.53, 9.63	<0.001
Outcome: Intubation						
No liver disease (REF)	1.00	—	—	1.00	—	—
NAALD no advanced fibrosis	1.65	1.51, 1.81	<0.001	1.52	1.38, 1.68	<0.001
NAALD cirrhosis	2.03	1.78, 2.32	<0.001	1.93	1.67, 2.22	<0.001
NAALD decompensated cirrhosis	11.08	10.12, 12.13	<0.001	9.83	8.90, 10.85	<0.001
ALD no advanced fibrosis	0.75	0.49, 1.14	0.173	1.08	0.69, 1.67	0.747
ALD cirrhosis	1.62	1.20, 2.19	0.002	1.63	1.19, 2.25	0.002
ALD decompensated cirrhosis	2.01	1.61, 2.52	<0.001	2.44	1.92, 3.10	<0.001
Alcohol‐associated hepatitis	2.48	1.61, 3.81	<0.001	2.90	1.84, 4.58	<0.001
Outcome: ICU admission						
No liver disease (REF)	1.00	—	—	1.00	—	—
NAALD no advanced fibrosis	1.48	1.37, 1.60	<0.001	1.40	1.29, 1.52	<0.001
NAALD cirrhosis	1.65	1.47, 1.86	<0.001	1.52	1.34, 1.72	<0.001
NAALD decompensated cirrhosis	7.44	6.79, 8.16	<0.001	6.30	5.72, 6.94	<0.001
ALD no advanced fibrosis	0.97	0.72, 1.32	0.866	1.21	0.88, 1.66	0.243
ALD cirrhosis	1.70	1.32, 2.19	<0.001	1.62	1.24, 2.11	<0.001
ALD decompensated cirrhosis	2.08	1.72, 2.53	<0.001	2.26	1.85, 2.76	<0.001
Alcohol‐associated hepatitis	2.66	1.82, 3.89	< 0.001	2.88	1.93, 4.29	<0.001

Most ALD categories were associated with higher odds of intubation and ICU admission, but again, this association was not as strong as in AH, nor comparable to NAALD categories. ALD in the absence of advanced fibrosis was not associated with a higher risk of intubation nor ICU admission compared to those with no liver disease. On the other hand, NAALD in the absence of advanced fibrosis was associated with a higher risk of intubation (aOR 1.52, 95% CI 1.38, 1.68, *p* < 0.001) and ICU admission (aOR 1.40, 1.29, 1.52, *p* < 0.001). ALD cirrhosis was associated with a higher risk of intubation (aOR 1.63, 95% CI 1.19, 2.25, *p* = 0.002) but again, not to the same degree as NAALD cirrhosis (aOR 1.93, 95% CI 1.67, 2.22, *p* < 0.001). ALD and NAALD cirrhosis carried similar risks for ICU admission (aOR 1.62, 95% CI 1.24, 2.11, *p* < 0.001) and (aOR 1.52, 95% CI 1.34, 1.72, *p* < 0.001), respectively. Finally, those with decompensated ALD cirrhosis had a higher risk of intubation (aOR 2.44, 95% CI 1.92, 3.10, *p* < 0.001) and ICU admission (aOR 2.26, 95% CI 1.85, 2.76, *p* < 0.001) but not as high as in those with AH (intubation aOR 2.90, 95% CI 1.84, 4.58, *p* < 0.001 and ICU admission aOR 2.88, 95% CI 1.93, 4.29, *p* < 0.001). Decompensated NAALD cirrhosis carried the highest risk of intubation (aOR 9.83, 95% CI 8.90, 10.85, *p* < 0.001) and ICU admission (aOR 6.30, 95% CI 5.72, 6.94, *p* < 0.001) compared to all ALD categories (Figure 2B,C).

Supplements 3–5 give complete results from the multivariate Approach #4 models. In addition to liver disease, male gender, increasing age, higher BMI, and former smoking status were all associated with increased mortality from COVID‐19, which is consistent with existing data. The use of remdesivir was associated with increased odds of mortality (aOR 1.30, 95% CI 1.25, 1.36, *p* < 0.001), intubation (aOR 1.58, 95% CI 1.52, 1.63, *p* < 0.001), and ICU admission (aOR 1.60, 95% CI 1.55, 1.65, *p* < 0.001). The use of steroids was also associated with increased odds of mortality (aOR 3.06, 95% CI 2.94, 3.18, *p* < 0.001), intubation (aOR 3.81, 95% CI 3.69, 3.95, *p* < 0.001), and ICU admission (aOR 2.61, 95% CI 2.54, 2.69, *p* < 0.001). Hospitalization after COVID‐19 vaccine availability was associated with decreased odds of mortality (aOR 0.76, 95% CI 0.72, 0.79, *p* < 0.001), intubation (aOR 0.77, 95% CI 0.74, 0.80, *p* < 0.001) and ICU admission (aOR 0.71, 95% CI 0.68, 0.73, *p* < 0.001) compared to hospitalization before COVID‐19 vaccine availability. In addition, receipt of 1 or more prehospital COVID‐19 vaccine doses was associated with decreased odds of all tested adverse outcomes (Supplements 3–5).

## DISCUSSION

Liver disease encompasses a broad spectrum of diagnoses from those with mild hepatitis and no advanced fibrosis to those with severe decompensated cirrhosis. In this multicenter study, we sought to examine how survival from COVID‐19 was impacted by history and severity of liver disease and if alcohol‐associated liver disease was an independent predictor of adverse outcomes.

Among 145,944 patients hospitalized with COVID‐19 across 21 health systems, a history of liver disease was associated with an increase in overall mortality during hospitalization as well as all adverse outcomes tested (Figure 2). As the severity of liver disease advanced from liver disease without significant fibrosis to decompensated cirrhosis, there was a progressive increase in the risk of all tested adverse outcomes including ICU admission, intubation, and death. These findings correlate with the known effect on morbidity and mortality of other infections occurring in patients with established liver disease (Bajaj et al., 2021). It also highlights the need for precautions to minimize the risk of exposure to COVID‐19 in patients with liver disease and the importance of vaccinating this population to mitigate the risk of COVID‐19.

Perhaps in response to the unique psychosocial stressors of a global pandemic, there has been a documented increase in alcohol consumption in the United States since the start of the COVID‐19 pandemic (Boschuetz et al., 2020; Leventhal et al., 2022). Other studies have shown a correlated increase in consequences associated with alcohol use including ALD and ALD mortality (Deutsch‐Link, Jiang, et al., 2022; Kulkarni et al., 2023; Pollard et al., 2020). We speculated that alcohol‐associated liver disease would correlate independently with adverse consequences of COVID‐19, above and beyond that from liver disease alone. However, our data did not confirm our prestudy hypothesis about the impact of alcohol. Instead, we found that although alcohol‐associated liver disease was associated with an increased risk of mortality, intubation, and ICU admission in patients with COVID‐19, the risk was less than that observed in patients with prior liver disease not related to alcohol. While decompensated cirrhosis increased the odds of mortality from COVID‐19 by an adjusted OR of 8.41, decompensated alcohol‐associated cirrhosis increased the odds of mortality by only 2.66 compared to those with no liver disease. In addition, independently, having an alcohol‐associated liver disease diagnosis was associated with reduced odds of death compared to having a nonalcohol‐associated liver disease with an adjusted OR of 0.30.

Our data do not provide an explanation for the paradoxical “protective” effect of an alcohol‐related diagnosis on the short‐term outcomes of COVID‐19. One possibility is that immune dysfunction in alcohol‐associated liver disease, and particularly recent consumption, acted to ameliorate the inflammatory responses to COVID‐19 (Minotti et al., 2020). Prior studies have demonstrated that alcohol consumption mediates innate and adaptive immune systems (Bailey et al., 2021; Barr et al., 2016; Chick, 2020; Szabo & Saha, 2015). In addition, several prospective studies have reported potentially favorable effects of alcohol consumption on COVID‐19 hospitalization as well as vulnerabilities to upper respiratory infections (Cohen, 2021; Hamer et al., 2020; Huang et al., 2022). We suspect that this is due to alcohol's immunomodulatory effect and association with reduced inflammatory cytokines and biomarkers of inflammation including C‐reactive protein (CRP) and interleukin‐6 (IL‐6) (Hamer et al., 2020; Szabo & Saha, 2015).

Regarding the impact of COVID‐19 on other at‐risk populations, such as males, former smokers, those of advancing age, and higher BMIs, our findings align with existing research that these populations are at higher risk for adverse outcomes (Bennett et al., 2021; Booth et al., 2021; Garg et al., 2020; Vardavas & Nikitara, 2020). In our study, the use of remdesivir and corticosteroids in the treatment of COVID‐19 was also associated with higher odds of all adverse clinical outcomes. This is likely due to confounding by clinical indication, with the sickest patients the most likely to receive treatment. Interestingly, patients with ALD were less likely to receive these treatments compared to those with NAALD. 22.7% of patients with ALD received remdesivir vs. 44.1% with NAALD, and 20.5% of patients with ALD received corticosteroids vs. 31.2% with NAALD. This may indicate that the ALD patients were less ill than the NAALD patients, potentially explaining the ALD patients' better outcomes overall.

This study also emphasizes the importance of COVID‐19 vaccination in patients with underlying liver disease. In all analyses, hospitalization after COVID‐19 vaccine availability was associated with less severe outcomes compared to hospitalization before COVID‐19 vaccine availability, as demonstrated in previous studies (Fiore et al., 2022). Likewise, Receipt of 1 or more prehospital COVID‐19 vaccine doses was associated with less severe outcomes, with a decreasing aOR with each dose (Baker et al., 2023). The Moderna and Pfizer‐BioNTech COVID‐19 vaccines are recommended for all patients 6 months and older, and the Novavax COVID‐19 vaccine for everyone 12 years and older. Multiple studies have demonstrated that patients with chronic liver disease and cirrhosis are at higher risk for adverse outcomes from COVID‐19 and should receive two doses of the 2024–2025 COVID‐19 vaccine 6 months apart.

This study is a large, multicenter study investigating the association between liver disease and alcohol‐associated liver disease and outcomes of patients hospitalized with COVID‐19. However, it does have several limitations. Due to the retrospective, observational nature of this study, we cannot confirm the causality of the associations reported, and we are unable to determine the degree of underlying liver fibrosis in those patients with liver disease. We are also unable to incorporate the expanding nomenclature regarding steatotic liver disease, including the combined clinical entity “MetALD” (Arab et al., 2025). In addition, underreporting of alcohol use due to stigma, the sick quitter effect, and recall bias could affect the ALD diagnosis, and disparities in healthcare access and patients lost to follow‐up could impact estimates of ALD‐related mortality in COVID‐19 (Schomerus et al., 2022). The absence of COVID‐negative patients also prevents us from determining whether adverse outcomes in patients with liver disease and COVID‐19 exceed those in liver disease patients in general. In addition, this study relies on the accuracy of ICD‐10 coding. Prior studies have demonstrated that complex coding rules have led to significant uncertainties in three‐and four‐digit ICD‐10 coding, which could potentially impact the outcomes of this study (Stausberg et al., 2008; Wockenfuss et al., 2009). However, the large number of patients included from multiple medical systems likely mitigates some of this coding uncertainty. Finally, our study was limited to outcomes during index hospitalization with COVID‐19. It does not address mortality or adverse outcomes of patients hospitalized with COVID‐19 following discharge. It is well known that some people previously infected with COVID‐19 experience long‐term effects known as “long COVID” weeks to years after infection, and these outcomes were not addressed in this study. Future studies might address how liver disease and alcohol‐associated liver disease impact outcomes related to long COVID.

## CONCLUSION

In this large, observational cohort of COVID‐19 patients, mortality in patients with liver disease was triple that seen in patients without liver disease. There was a clear gradient of adverse outcomes across the severity of liver disease, and nonalcohol forms of liver disease carried higher odds of adverse outcomes compared to alcohol‐associated liver disease across all severities.

## CONFLICT OF INTEREST STATEMENT

The authors declare that there are no conflicts of interest related to the research presented in this manuscript.

## Supporting information


Appendix S1

## Data Availability

The data that support the findings of this study are available from the corresponding author upon reasonable request.
