# Supplementary material for: Alcohol‐associated liver disease is associated with less adverse outcomes compared to nonalcohol‐associated liver disease in patients with COVID‐19
Source: Alcohol Clin Exp Res (Hoboken). 2025 Jul 30;49(9):1983–92. doi: 10.1111/acer.70124 (PMC12463747; doi:10.1111/acer.70124)
Supplement: Supplementary file 1 — Appendix S1 [file ACER-49-1983-s001.docx]

**Supplements**

**Supplement 1:** ICD-10 Codes by Group

**AUD with liver disease:**

K70 Alcoholic liver disease

K70.0 Alcoholic fatty liver

K70.2 Alcoholic fibrosis and sclerosis of liver

K70.9 Alcoholic liver disease unspecified

K70.4 Alcoholic hepatic failure

K70.40 Alcoholic hepatic failure without coma

**No AUD with liver disease:**

K74.0 Hepatic fibrosis

K74.1 Hepatic sclerosis

K74.2 Hepatic fibrosis with hepatic sclerosis

K72 Hepatic failure, not elsewhere classified

K72.0 Acute and subacute hepatic failure

K72.00 Acute and subacute hepatic failure without coma

K72.01 Acute and subacute hepatic failure with coma

K75 Other inflammatory liver diseases

K75.2 Nonspecific reactive hepatitis

K75.3 Granulomatous hepatitis, not elsewhere classified

K75.4 Autoimmune hepatitis

K75.8 Other specified inflammatory liver disease

K75.81 Nonalcohol steatohepatitis (NASH)

K75.89 Other specified inflammatory liver diseases

K75.9 Inflammatory liver disease, unspecified

K76.0 Fatty change of liver, not elsewhere classified

K83.01 Primary sclerosing cholangitis

K83.09 Other cholangitis

**AUD with cirrhosis:**

K70.3 Alcoholic cirrhosis of liver

K70.30 Alcoholic cirrhosis of liver without ascites

**No AUD with cirrhosis:**

K72.1 Chronic hepatic failure

K72.10 Chronic hepatic failure without coma

K72.9 Hepatic failure, unspecified

K72.90 Hepatic failure, unspecified without coma

K74 Fibrosis and cirrhosis of liver

K74.4 Secondary biliary cirrhosis

K74.5 Biliary cirrhosis, unspecified

K74.6 Other and unspecified cirrhosis of liver

K74.69 Other cirrhosis of liver

**AUD with decompensation:**

K70.31 Alcoholic cirrhosis of liver with ascites

K70.41 Alcoholic hepatic failure with coma

**No AUD with decompensation:**

K72.0 Acute and subacute hepatic failure without coma

K72.11 Chronic hepatic failure with coma

K72.91 Hepatic failure unspecified, with coma

K76.7 Hepatorenal syndrome

K76.6 Portal hypertension

R18.9 Other ascites

I85.01 Esophageal varices with bleeding

**AH:**

K70.1 Alcoholic hepatitis

K70.11 Alcoholic hepatitis with ascites

**Supplement 2:** Patient characteristics with liver disease cases subclassified as non-alcohol-associated liver disease (NAALD) and alcohol-associated liver disease (ALD).

|  | **Total**  **(N = 145,944)** | **No Liver**  **Disease**  **(N = 137,993)** | **Non-Alcohol- Associated Liver Disease**  **(N = 6,798)** | **Alcohol-Associated Liver Disease**  **(N = 1,153)** |
| --- | --- | --- | --- | --- |
| **Sex** |  |  |  |  |
| Female | 74,538 (51.1%) | 71,064 (51.5%) ^a^ | 3,151 (46.4%) ^b^ | 323 (28.0%) ^c^ |
| Male | 71,402 (48.9%) | 66,925 (48.5%) ^a^ | 3,647 (53.6% )^b^ | 830 (72.0%) ^c^ |
| Other | 4 (0.0%) | 4 (0.0%) | 0 (0.0%) | 0 (0.0%) |
| **Age Group** |  |  |  |  |
| 18-29 | 9,871 (6.8%) | 9,554 (6.9% )^a^ | 290 (4.3%) ^b^ | 27 (2.3%) ^c^ |
| 30-39 | 13,051 (8.9%) | 12,389 (9.0% )^a^ | 512 (7.5%) ^b^ | 150 (13.0%) ^c^ |
| 40-49 | 14,666 (10.0%) | 13,616 (9.9%) ^a^ | 845 (12.4%) ^b^ | 205 (17.8%) ^c^ |
| 50-64 | 39,610 (27.1%) | 36,632 (26.5%) ^a^ | 2,481 (36.5%) ^b^ | 497 (43.1%) ^c^ |
| 65-74 | 29,868 (20.5%) | 28,089 (20.4%) ^a^ | 1,576 (23.2%) ^b^ | 203 (17.6%) ^c^ |
| 75-84 | 23,984 (16.4%) | 23,101 (16.7%) ^a^ | 823 (12.1%) ^b^ | 60 (5.2%) ^c^ |
| 85+ | 14,894 (10.2%) | 14,612 (10.6%) ^a^ | 271 (4.0%) ^b^ | 11 (1.0%) ^c^ |
| **Race** |  |  |  |  |
| American Indian or Alaska Native | 546 (0.4%) | 485 (0.4%) ^a^ | 42 (0.6%) ^b^ | 19 (1.6%) ^c^ |
| Asian | 3,882 (2.7%) | 3,674 (2.7%) ^a^ | 193 (2.8%) ^a^ | 15 (1.3%) ^b^ |
| Black or African American | 34,663 (23.8%) | 33,161 (24.0%) ^a^ | 1,293 (19.0%) ^b^ | 209 (18.1% ) ^b^ |
| Native Hawaiian or Other Pacific Islander | 588 (0.4%) | 549 (0.4%) ^a^ | 36 (0.5%) ^a^ | 3 (0.3%) ^a^ |
| White | 85,851 (58.8%) | 80,953 (58.7%) ^a^ | 4,191 (61.7%) ^b^ | 707 (61.3%) ^a, b^ |
| Other Race Not Specified | 17,384 (11.9%) | 16,314 (11.8%) ^a^ | 889 (13.1%) ^b^ | 181 (15.7%) ^c^ |
| More than One Race | 571 (0.4%) | 533 (0.4%) ^a^ | 32 (0.5%) ^a^ | 6 (0.5%) ^a^ |
| Unknown, Not Reported, or Missing | 2,459 (4.7%) | 2,324 (1.7%) ^a^ | 122 (1.8%) ^a^ | 13 (1.1%) ^a^ |
| **Ethnicity** |  |  |  |  |
| Not Hispanic or Latino | 120,761 (82.7%) | 114,435 (82.9%) ^a^ | 5,432 (79.9%) ^b^ | 894 (77.5%) ^b^ |
| Hispanic or Latino | 22,373 (15.3%) | 20,914 (15.2%) ^a^ | 1,214 (17.9%) ^b^ | 245 (21.2%) ^c^ |
| Unknown, Not Reported, or Missing | 2,810 (1.9%) | 2,644 (1.9%) ^a, b^ | 152 (2.2%) ^b^ | 14 (1.2%) ^c^ |
| **BMI** |  |  |  |  |
| Underweight | 4,504 (3.1%) | 4,300 (3.1%) ^a^ | 147 (2.2%) ^b^ | 382 (33.1%) ^c^ |
| Healthy Weight | 33,608 (23.0%) | 31,978 (23.2%) ^a^ | 1,248 (18.4%) ^b^ | 57 (4.9%) ^c^ |
| Overweight | 41,473 (28.4%) | 39,390 (28.5%) ^a^ | 1,730 (25.4%) ^b^ | 353 (30.6%) ^a^ |
| Obese | 48,138 (33.0%) | 45,306 (32.8%) ^a^ | 2,534 (37.3%) ^b^ | 298 (25.8% ^c^) |
| Severely Obese | 16,627 (11.4%) | 15,482 (11.2%) ^a^ | 1,085 (16.0%) ^b^ | 60 (5.2%) ^c^ |
| Missing or Biologically Implausible | 1,594 (1.1%) | 1,537 (1.1%) ^a^ | 54 (0.8%) ^b^ | 3 (0.3%) ^c^ |
| **Vaccine Availability at Hospitalization** |  |  |  |  |
| Before Vaccines Available | 50,433 (34.6%) | 47,875 (34.7%) ^a^ | 2,213 (32.6%) ^b^ | 345 (29.9%) ^b^ |
| After Vaccines Available | 95,511 (65.4%) | 90,118 (65.3%) ^a^ | 4,585 (67.4%) ^b^ | 808 (70.1%) ^b^ |
| **Pre-Hospitalization Vaccine Doses** |  |  |  |  |
| 0 | 123,126 (84.4%) | 116,525 (84.4%) ^a^ | 5,693 (83.7%) ^a^ | 908 (78.8%) ^b^ |
| 1 | 5,589 (3.8%) | 5,219 (3.8%) ^a^ | 293 (4.3%) ^b^ | 77 (6.7%) ^c^ |
| 2 | 13,651 (9.4%) | 12,867 (9.3%) ^a^ | 631 (9.3%) ^a^ | 153 (13.3%) ^b^ |
| 3 | 3,578 (2.5%) | 3,382 (2.5%) ^a^ | 181 (2.7%) ^a^ | 15 (1.3%) ^b^ |
| *Note:* Groups in a row that do not share the same letter superscript differ from one another on the row variable at *p* < .05 | | | | |

| **Supplement 3:** Full multivariate model predicting mortality by severity of liver disease in alcohol-associated liver disease (ALD) vs non-alcohol-associated liver disease (NAALD) vs alcohol-associated hepatitis (AH) at index COVID-19 hospitalization | | | | | | | |
| --- | --- | --- | --- | --- | --- | --- | --- |
| **Predictor** | | **aOR** | | **95% CI** | | **p** | |
| **Liver Disease** | |  | |  | |  | |
| None (REF) | | 1.00 | | -- | | -- | |
| Non-Alcohol Liver Disease/Fibrosis | | 1.40 | | 1.23, 1.59 | | <.001 | |
| Non-Alcohol Cirrhosis | | 2.22 | | 1.91, 2.58 | | <.001 | |
| Non-Alcohol Decompensated Cirrhosis | | 10.21 | | 9.24, 11.28 | | <.001 | |
| Alcohol Liver Disease/Fibrosis | | 0.66 | | 0.31, 1.41 | | .279 | |
| Alcohol Cirrhosis | | 1.31 | | 0.88, 1.97 | | .188 | |
| Alcohol Decompensated Cirrhosis | | 2.66 | | 2.02, 3.51 | | <.001 | |
| Alcohol-Associated Hepatitis | | 5.83 | | 3.53, 9.63 | | <.001 | |
| **Sex** | |  | |  | |  | |
| Female (REF) | | 1.00 | | -- | | -- | |
| Male | | 1.50 | | 1.44, 1.56 | | <.001 | |
| **Age** | |  | |  | |  | |
| 18 to 29 (REF) | | 1.00 | | -- | | -- | |
| 30 to 39 | | 1.52 | | 1.19, 1.95 | | <.001 | |
| 40 to 49 | | 2.82 | | 2.25, 3.53 | | <.001 | |
| 50 to 64 | | 5.60 | | 4.53, 6.92 | | <.001 | |
| 65 to 74 | | 8.62 | | 6.92, 10.74 | | <.001 | |
| 75 to 84 | | 13.21 | | 10.60, 16.47 | | <.001 | |
| 85+ | | 19.62 | | 15.70, 24.52 | | <.001 | |
| **Race** | |  | |  | |  | |
| White (REF) | | 1.00 | | -- | | -- | |
| American Indian or Alaska Native | | 1.83 | | 1.39, 2.41 | | <.001 | |
| Asian | | 1.53 | | 1.37, 1.71 | | <.001 | |
| Black or African American | | 1.10 | | 1.04, 1.16 | | <.001 | |
| Native Hawaiian or Pacific Islander | | 1.07 | | 0.77, 1.49 | | .679 | |
| Other Race Not Specified | | 1.18 | | 1.09, 1.28 | | <.001 | |
| More than One | | 1.39 | | 1.00, 1.92 | | .051 | |
| Unknown or Missing | | 1.31 | | 1.12, 1.52 | | <.001 | |
| **Ethnicity** | |  | |  | |  | |
| Not Hispanic or Latino (REF) | | 1.00 | | -- | | -- | |
| Hispanic or Latino | | 1.14 | | 1.06, 1.23 | | <.001 | |
| Missing | | 1.25 | | 1.09, 1.43 | | .002 | |
| **Insurance Status** | |  | |  | |  | |
| Commercial (REF) | | 1.00 | | -- | | -- | |
| Medicare | | 1.28 | | 1.18, 1.39 | | <.001 | |
| Medicaid | | 1.11 | | 1.01, 1.22 | | .032 | |
| Commercial | | 1.68 | | 1.48, 1.91 | | <.001 | |
| Other | | 1.12 | | 1.01, 1.24 | | .041 | |
| **Smoking Status** | |  | |  | |  | |
| Never Smoker (REF) | | 1.00 | | -- | | -- | |
| Current Smoker | | 0.94 | | 0.86, 1.03 | | .164 | |
| Former Smoker | | 1.07 | | 1.03, 1.12 | | .002 | |
| Missing Smoking Status | | 1.05 | | 0.97, 1.13 | | .231 | |
| **BMI** | |  | |  | |  | |
| Healthy Weight (REF) | | 1.00 | | -- | | -- | |
| Underweight | | 1.22 | | 1.10, 1.36 | | <.001 | |
| Overweight | | 1.13 | | 1.07, 1.19 | | <.001 | |
| Obese | | 1.33 | | 1.26, 1.41 | | <.001 | |
| Severely Obese | | 1.95 | | 1.81, 2.10 | | <.001 | |
| Missing or Biologically Implausible | | 1.97 | | 1.69, 2.30 | | <.001 | |
| **Remdesivir** During Hospitalization | |  | |  | |  | |
| No (REF) | | 1.00 | | -- | | -- | |
| Yes | | 1.30 | | 1.25, 1.36 | | <.001 | |
| **Steroid Medication** During Hospitalization | |  | |  | |  | |
| No (REF) | | 1.00 | | -- | | -- | |
| Yes | | 3.06 | | 2.94, 3.18 | | <.001 | |
| **Hospitalized After Vaccines Available** | |  | |  | |  | |
| No (REF) | | 1.00 | | -- | | -- | |
| Yes | | 0.76 | | 0.72, 0.79 | | <.001 | |
| **Pre-Hospital Vaccine Doses** | |  | |  | |  | |
| 0 (REF) | | 1.00 | | -- | | -- | |
| 1 | | 0.59 | | 0.52, 0.67 | | <.001 | |
| 2 | | 0.61 | | 0.56, 0.65 | | <.001 | |
| 3 | | 0.49 | | 0.42, 0.56 | | <.001 | |
| **Elixhauser Comorbidity Index** | | 1.012 | | 1.010, 1.014 | | < .001 | |
| **Supplement 4:** Full multivariate model predicting intubation by severity of liver disease in alcohol-associated liver disease (ALD) vs non-alcohol-associated liver disease (NAALD) vs alcohol-associated hepatitis (AH) at index COVID-19 hospitalization | | | | | | | |
| **Predictor** | | **aOR** | | **95% CI** | | **p** | |
| **Liver Disease** | |  | |  | |  | |
| None (REF) | | 1.00 | | -- | | -- | |
| Non-Alcohol Liver Disease/Fibrosis | | 1.52 | | 1.38, 1.68 | | <.001 | |
| Non-Alcohol Cirrhosis | | 1.93 | | 1.67, 2.22 | | <.001 | |
| Non-Alcohol Decompensated Cirrhosis | | 9.83 | | 8.90, 10.85 | | <.001 | |
| Alcohol Liver Disease/Fibrosis | | 1.08 | | 0.69, 1.67 | | .747 | |
| Alcohol Cirrhosis | | 1.63 | | 1.19, 2.25 | | .002 | |
| Alcohol Decompensated Cirrhosis | | 2.44 | | 1.92, 3.10 | | <.001 | |
| Alcohol-Associated Hepatitis | | 2.90 | | 1.84, 4.58 | | <.001 | |
| **Sex** | |  | |  | |  | |
| Female (REF) | | 1.00 | | -- | | -- | |
| Male | | 1.59 | | 1.54, 1.65 | | <.001 | |
| **Age** | |  | |  | |  | |
| 18 to 29 (REF) | | 1.00 | | -- | | -- | |
| 30 to 39 | | 1.06 | | 0.94, 1.19 | | .334 | |
| 40 to 49 | | 1.50 | | 1.35, 1.67 | | <.001 | |
| 50 to 64 | | 2.07 | | 1.88, 2.28 | | <.001 | |
| 65 to 74 | | 2.30 | | 2.06, 2.56 | | <.001 | |
| 75 to 84 | | 2.16 | | 1.93, 2.41 | | <.001 | |
| 85+ | | 1.46 | | 1.30, 1.65 | | <.001 | |
| **Race** | |  | |  | |  | |
| White (REF) | | 1.00 | | -- | | -- | |
| American Indian or Alaska Native | | 1.56 | | 1.23, 1.98 | | <.001 | |
| Asian | | 1.54 | | 1.40, 1.70 | | <.001 | |
| Black or African American | | 1.12 | | 1.07, 1.17 | | <.001 | |
| Native Hawaiian or Pacific Islander | | 1.06 | | 0.83, 1.36 | | .635 | |
| Other Race Not Specified | | 1.19 | | 1.11, 1.27 | | <.001 | |
| More than One | | 1.58 | | 1.25, 2.01 | | <.001 | |
| Unknown or Missing | | 1.28 | | 1.13, 1.46 | | <.001 | |
| **Ethnicity** | |  | |  | |  | |
| Not Hispanic or Latino (REF) | | 1.00 | | -- | | -- | |
| Hispanic or Latino | | 1.08 | | 1.01, 1.15 | | .017 | |
| Missing | | 1.30 | | 1.16, 1.46 | | <.001 | |
| **Insurance Status** | |  | |  | |  | |
| Commercial (REF) | | 1.00 | | -- | | -- | |
| Medicare | | 1.25 | | 1.18, 1.33 | | <.001 | |
| Medicaid | | 1.26 | | 1.19, 1.35 | | <.001 | |
| Commercial | | 1.30 | | 1.15, 1.45 | | <.001 | |
| Other | | 1.14 | | 1.06, 1.23 | | <.001 | |
| **Smoking Status** | |  | |  | |  | |
| Never Smoker (REF) | | 1.00 | | -- | | -- | |
| Current Smoker | | 1.03 | | 0.96, 1.10 | | .427 | |
| Former Smoker | | 1.07 | | 1.03, 1.11 | | .001 | |
| Missing Smoking Status | | 1.71 | | 1.60, 1.82 | | <.001 | |
| **BMI** | |  | |  | |  | |
| Healthy Weight (REF) | | 1.00 | | -- | | -- | |
| Underweight | | 0.99 | | 0.88, 1.10 | | .801 | |
| Overweight | | 1.09 | | 1.04, 1.14 | | <.001 | |
| Obese | | 1.40 | | 1.33, 1.47 | | <.001 | |
| Severely Obese | | 2.16 | | 2.04, 2.30 | | <.001 | |
| Missing or Biologically Implausible | | 0.96 | | 0.80, 1.14 | | .617 | |
| **Remdesivir** During Hospitalization | |  | |  | |  | |
| No (REF) | | 1.00 | | -- | | -- | |
| Yes | | 1.58 | | 1.52, 1.63 | | <.001 | |
| **Steroid Medication** During Hospitalization | |  | |  | |  | |
| No (REF) | | 1.00 | | -- | | -- | |
| Yes | | 3.81 | | 3.69, 3.95 | | <.001 | |
| **Hospitalized After Vaccines Available** | |  | |  | |  | |
| No (REF) | | 1.00 | | -- | | -- | |
| Yes | | 0.77 | | 0.74, 0.80 | | <.001 | |
| **Pre-Hospital Vaccine Doses** | |  | |  | |  | |
| 0 (REF) | | 1.00 | | -- | | -- | |
| 1 | | 0.76 | | 0.69, 0.84 | | <.001 | |
| 2 | | 0.71 | | 0.66, 0.75 | | <.001 | |
| 3 | | 0.67 | | 0.59, 0.75 | | <.001 | |
| **Elixhauser Comorbidity Index** | | 0.997 | | 0.995, 0.999 | | < .001 | |
| **Supplement 5:** Full multivariate model predicting ICU admission by severity of liver disease in alcohol-associated liver disease (ALD) vs non-alcohol-associated liver disease (NAALD) vs alcohol-associated hepatitis (AH) at index COVID-19 hospitalization | | | | | | | |
| **Predictor** | | **aOR** | | **95% CI** | | **p** | |
| **Liver Disease** | |  | |  | |  | |
| None (REF) | | 1.00 | | -- | | -- | |
| Non-Alcohol Liver Disease/Fibrosis | | 1.40 | | 1.29, 1.52 | | <.001 | |
| Non-Alcohol Cirrhosis | | 1.52 | | 1.34, 1.72 | | <.001 | |
| Non-Alcohol Decompensated Cirrhosis | | 6.3 | | 5.72, 6.94 | | <.001 | |
| Alcohol Liver Disease/Fibrosis | | 1.21 | | 0.88, 1.66 | | .243 | |
| Alcohol Cirrhosis | | 1.62 | | 1.24, 2.11 | | <.001 | |
| Alcohol Decompensated Cirrhosis | | 2.26 | | 1.85, 2.76 | | <.001 | |
| Alcohol-Associated Hepatitis | | 2.88 | | 1.93, 4.29 | | <.001 | |
| **Sex** | |  | |  | |  | |
| Female (REF) | | 1.00 | | -- | | -- | |
| Male | | 1.43 | | 1.39, 1.47 | | <.001 | |
| **Age** | |  | |  | |  | |
| 18 to 29 (REF) | | 1.00 | | -- | | -- | |
| 30 to 39 | | 0.97 | | 0.90, 1.06 | | .525 | |
| 40 to 49 | | 1.28 | | 1.18, 1.37 | | <.001 | |
| 50 to 64 | | 1.51 | | 1.41, 1.61 | | <.001 | |
| 65 to 74 | | 1.62 | | 1.50, 1.75 | | <.001 | |
| 75 to 84 | | 1.54 | | 1.42, 1.67 | | <.001 | |
| 85+ | | 1.11 | | 1.02, 1.22 | | .017 | |
| **Race** | |  | |  | |  | |
| White (REF) | | 1.00 | | -- | | -- | |
| American Indian or Alaska Native | | 1.39 | | 1.14, 1.69 | | .001 | |
| Asian | | 1.21 | | 1.11, 1.32 | | <.001 | |
| Black or African American | | 1.03 | | 0.99, 1.07 | | .109 | |
| Native Hawaiian or Pacific Islander | | 0.95 | | 0.76, 1.18 | | .640 | |
| Other Race Not Specified | | 1.07 | | 1.01, 1.14 | | .016 | |
| More than One | | 1.45 | | 1.18, 1.77 | | <.001 | |
| Unknown or Missing | | 1.15 | | 1.03, 1.28 | | .015 | |
| **Ethnicity** | |  | |  | |  | |
| Not Hispanic or Latino (REF) | | 1.00 | | -- | | -- | |
| Hispanic or Latino | | 1.03 | | 0.98, 1.09 | | .250 | |
| Missing | | 1.29 | | 1.16, 1.43 | | <.001 | |
| **Insurance Status** | |  | |  | |  | |
| Commercial (REF) | | 1.00 | | -- | | -- | |
| Medicare | | 1.13 | | 1.07, 1.19 | | <.001 | |
| Medicaid | | 1.13 | | 1.07, 1.19 | | <.001 | |
| Commercial | | 1.19 | | 1.09, 1.29 | | <.001 | |
| Other | | 1.16 | | 1.10, 1.24 | | <.001 | |
| **Smoking Status** | |  | |  | |  | |
| Never Smoker (REF) | | 1.00 | | -- | | -- | |
| Current Smoker | | 1.03 | | 0.97, 1.08 | | .335 | |
| Former Smoker | | 1.03 | | 1.00, 1.06 | | .064 | |
| Missing Smoking Status | | 1.58 | | 1.50, 1.67 | | <.001 | |
| **BMI** | |  | |  | |  | |
| Healthy Weight (REF) | | 1.00 | | -- | | -- | |
| Underweight | | 1.00 | | 0.92, 1.09 | | .995 | |
| Overweight | | 0.97 | | 0.94, 1.01 | | .152 | |
| Obese | | 1.07 | | 1.03, 1.11 | | <.001 | |
| Severely Obese | | 1.38 | | 1.31, 1.45 | | <.001 | |
| Missing or Biologically Implausible | | 0.82 | | 0.71, 0.94 | | .004 | |
| **Remdesivir** During Hospitalization | |  | |  | |  | |
| No (REF) | | 1.00 | | -- | | -- | |
| Yes | | 1.60 | | 1.55, 1.65 | | <.001 | |
| **Steroid Medication** During Hospitalization | |  | |  | |  | |
| No (REF) | | 1.00 | | -- | | -- | |
| Yes | | 2.61 | | 2.54, 2.69 | | <.001 | |
| **Hospitalized After Vaccines Available** | |  | |  | |  | |
| No (REF) | | 1.00 | | -- | | -- | |
| Yes | | 0.71 | | 0.68, 0.73 | | <.001 | |
| **Pre-Hospital Vaccine Doses** | |  | |  | |  | |
| 0 (REF) | | 1.00 | | -- | | -- | |
| 1 | | 0.85 | | 0.78, 0.91 | | <.001 | |
| 2 | | 0.82 | | 0.77, 0.86 | | <.001 | |
| 3 | | 0.77 | | 0.70, 0.85 | | <.001 | |
| **Elixhauser Comorbidity Index** | | 1.001 | | 0.999, 1.002 | | .412 | |
